# 

**Published:** 1892-09

**Authors:** 


ESTABLISHED 1855. SUBSCRIPTION, $2 00.

### ATLANTA MEDICAL AND SURGICAL JOURNAL.

SURGEON GENL'S OFFICE 147497 LIBRARY

OLD SERIES, VOL. XXVI. } NEW SERIES, VOL. IX. } ATLANTA, GA., SEPTEMBER, 1892. NO. 7.

LUTHER B. GRANDY, M. D., MANAGING EDITOR.

M. B. HUTCHINS, M. D. BUSINESS MANAGER.

